# Protective vaccinations in the control and prevention of infectious diseases—knowledge of adult Poles in this field. Preliminary results

**DOI:** 10.1186/s12889-022-14821-2

**Published:** 2022-12-14

**Authors:** Józefa Dąbek, Oskar Sierka, Zbigniew Gąsior

**Affiliations:** 1 Department of Cardiology, Faculty of Health Sciences in Katowice, Medical University of Silesia in Katowice, Ziołowa Street 45/47, 40-635 Katowice, Poland; 2 Student Research Group at the Department of Cardiology, Faculty of Health Sciences in Katowice, Medical University of Silesia in Katowice, Ziołowa Street 45/47, 40-635 Katowice, Poland

**Keywords:** Protective vaccinations, Knowledge, Adult Poles

## Abstract

**Background:**

Protective vaccinations are one of the basic means of infectious disease prevention. The aim of the study was to assess the implementation of compulsory and additional protective vaccinations among adult Poles, their knowledge about the purpose of introducing a vaccination schedule and adverse events following immunization. Opinions about and support for anti-vaccination movements were also examined.

**Materials and methods:**

A total of 700 (100%) people aged 18 to 80 (x̅  = 32,16 ± 16,46) took part in the study, conducted using the proprietary questionnaire. All the participants selected randomly from patients of the Department of Cardiology, their visitors, doctors, nurses, paramedics, medical students, and authors’ acquaintances gave their informed consent to participate in the study.

**Results:**

About 10% of the respondents did not complete the compulsory vaccination schedule. Almost 80% of respondents believed that the main reason for vaccinations schedule occurrence was the desire to completely exclude certain diseases and their complications from the population. More than half of the respondents have never had any additional vaccination. A statistically significant correlation was found between intake of at least one additional vaccination and age (*χ*^2^=22.262, *p*** = **0.002) and education level (*χ*^2^= 11.074, *p* = 0.004). Among the respondents, there was a group that classified autism as one of the adverse events following immunization. About 95% of respondents never experienced any adverse events following immunizationand as many as 30 respondents declared their support for anti-vaccination movements.

**Conclusion:**

The degree of the implementation of compulsory protective vaccinations in the study group was high, while additional vaccinations were insufficient. The purposefulness of introducing a vaccination schedule was correctly identified by the majority of the respondents, but the knowledge about adverse events following immunisation and their types was incomplete. Among participants were individuals who declared their support for anti-vaccination movements, so society should be constantly educated about vaccinations benefits.

**Supplementary Information:**

The online version contains supplementary material available at 10.1186/s12889-022-14821-2.

## Background

Protective vaccinations, along with the discovery of antibiotics, are considered an important achievement of modern medicine. Their introduction and application contributed to the control of various diseases (e. g. smallpox) and a radical reduction in the number of others (e. g. poliomyelitis), which were a scourge of humanity before the vaccination era, fearful all over the world due to its huge number of complications and deaths. In previous centuries, it was already noticed that people who suffered from a given disease often became resistant to it, but it was not until the eighteenth century that breakthrough discoveries related to the discussed topic were made. In 1796, Edward Jenner experimented with an eight-year-old boy who was vaccinated with the Vaccinia virus. Thanks to this procedure, the boy also became immune to smallpox. From that moment on, Jenner's vaccination procedure began its expansion not only in Europe but also in the world [1].

The beginnings of compulsory vaccination attempts can be traced back to nineteenth century France, wherein in 1805 Marianne Elisa de Lucca (Napoleon Bonaparte's sister) tried to introduce them to the population. However, she was unable to define a practical method of enforcing their execution. The aforementioned problem was solved by the inhabitants of the United Kingdom, in which "The United Kingdom Vaccination Act" of 1853 is considered to be the first vaccination schedule and, at the same time, a set of vaccinations that were compulsory at that time. It stipulates that smallpox vaccination is compulsory during the first three months of the life of an infant. The penalty for a parent who did not comply with the aforementioned act was a fine or imprisonment [2]. The first vaccination schedule in Poland dates back to the 1960s, when the Protective Vaccination Program was introduced, divided into compulsory and recommended vaccinations. Since then, it has been continuously developed and improved, taking into account access to new vaccines and scientific evidence of their effectiveness.

Despite such a long history of vaccinations, smallpox, which had plagued mankind for many centuries, was not eradicated from the world's population until 1980 [3]. Currently, vaccinologists are working on improving existing vaccines and developing new ones. Correctly performed vaccination is completely safe, however, as in any medical procedure, there is a risk of complications of varying severity.

Adverse events following immunization/vaccination (AEFI/AEFV) are any health disorder that occurs within 4 weeks after vaccine administration, or longer in the case of tuberculosis vaccination, and are the result of a manufacturing defect or individual patient reaction [4]. The most common adverse events after vaccination are mild local and general events such as swelling, inflammation at the site of vaccine injection, fever, and malaise. During the qualification for vaccination, patients should be informed that any symptoms, classified by them as AEFIs, do not have to be related to the vaccination itself, but also to other events causing the symptoms reported by them occurring in the post-vaccination period (a time relationship, not a cause-effect relationship). [5].

According to the Announcement of the Chief Sanitary Inspector, Polish compulsory vaccinations in 2022 include vaccination against tuberculosis, hepatitis B, rotaviruses, diphtheria, pertussis, tetanus, *Haemophilus influenzae* type b, *Pneumococci*, and vaccination against measles, mumps, and rubella. The obligatory vaccines are intended mostly for newborns and children. Within 24 h after birth, the healthy newborn should receive a subcutaneous vaccine against tuberculosis and an intramuscular or subcutaneous (according to the manufacturer's instructions) vaccine against type B hepatitis. In the second month of life (after the age of 6 weeks), the first dose of the rotavirus’s vaccine and the first dose of diphtheria, tetanus, and pertussis vaccine (combined vaccine) along with the second dose of type B hepatitis vaccine is administered. During the mentioned period, the first dose of vaccine against invasive *Haemophilus influenzae* type b, and invasive *Streptococcus pneumoniae* infections are also administered. At 4 months of age, the first dose of *poliomyelitis* vaccination, as well as the second dose of vaccination against diphtheria, pertussis, tetanus (combined vaccine), rotaviruses, invasive *Haemophilus influenzae* type b, and invasive *Streptococcus pneumoniae* infections are administered. At 5–6 months, the child should receive a second dose of vaccination against *poliomyelitis* and the third dose of vaccine against diphtheria, pertussis, tetanus (combined vaccine), rotaviruses*,* and invasive *Haemophilus influenzae* type b infections. At 7 months, the third dose of the type B hepatitis vaccine is administered. At 13–15 months, a combined measles, mumps, and rubella vaccine, along with the third dose of vaccine against invasive *Streptococcus pneumoniae* infections, *poliomyelitis*, and the fourth dose of a combined vaccine against diphtheria, pertussis, tetanus, and vaccine against invasive *Haemophilus influenzae* type b infections are administered. At this age, primary vaccinations are completed.

Further vaccinations, despite their booster nature, are included in the Polish vaccination schedule as an integral part of the prevention of infectious diseases. Booster vaccinations include those against diphtheria, pertussis, tetanus (one booster vaccination spread over time in 3 doses administered at different ages), measles, mumps, rubella, and poliomyelitis. At age 6, the child receives the first dose of booster vaccinations against diphtheria, pertussis, tetanus (combined vaccine) measles, mumps, rubella (combined vaccine), and p*oliomyelitis*. At age 10, a booster vaccination against measles, mumps, and rubella is administered. At age 14 the second dose of booster vaccination against diphtheria, pertussis, and tetanus is given to the children and the last compulsory vaccination is against diphtheria, and tetanus (third dose of booster vaccination) - administered at the age of 19. The above description refers to the standard schedule composed of the most common and refunded by the Polish National Health Fund vaccines. However, it should be noted that other vaccination schedules are available with self-paid vaccines. It also should not be forgotten that basic vaccinations for society members who, for some reason, have not completed the full vaccination schedule, become recommended vaccinations which, in addition to those described above, include vaccinations against influenza, *meningococci*, human papillomavirus, *Varicella zoster* virus, type A hepatitis, tick-borne encephalitis, cholera, typhoid fever, yellow fever, and rabies. Those additional vaccinations can also be administered to children, taking into account the recommendations contained in the annual announcement of the Polish Chief Sanitary Inspector about the Annual Immunization Program and the dosage described in the leaflet provided along with the vaccine by its manufacturer. All received vaccinations are recorded in the immunization record kept by the family doctor's clinic and in the child's health booklet, which covers all medical procedures performed up to the age of 18. The last vaccination given at age 19 is also recorded in the mentioned booklet [6].

The intensification of activity and support for anti-vaccination movements in recent years, which already existed in the times of Edward Jenner, is a cause of great concern. Their members more than once raise arguments against vaccinations which are completely inconsistent with reality. They cite evidence that does not support their claims of the "harmfulness" of vaccines. Many people promoting the ideas of these movements during the SARS-CoV-2 virus pandemic openly supported conspiracy theories that pharmaceutical companies wanted to control all those vaccinated. The ideas propagated by members of the anti-vaccination movement are dangerous and distort the overall beneficial picture of vaccinations. Once heard, they are hard to explain to a non-medically related citizens, according to the saying of the American Nobel laureate Saul Bellow—"A fool can throw a stone into a pond that 100 wise people will not be able to pull out." The aim of the study was to assess the implementation of compulsory and additional preventive vaccinations by the adult Poles, their knowledge about the purpose of vaccination schedule introduction and adverse events following immunization as well as their opinions on anti-vaccination movements.

## Materials and methods

Total of 700 people (100%) aged 18 to 80 years, (x̅  = 32,16 ± 16,46) were surveyed. The majority of the participants were women (500; 71.43%). The presented topic was part of a large project entitled "Medical knowledge of non-professionals as a predictor of health behaviour" submitted to the Bioethics Committee at the Medical University of Silesia in Katowice. In the justification of the opinion, by the decision from 17.10.2017 (KNW/0022/KB/223/17), the Bioethics Committee stated that the research based on a survey is not a medical experiment and that neither the evaluation nor the ethical approval of the committee is required to conduct it. Nevertheless, all participants declared their informed consent to participate in this study. The study was conducted using the proprietary questionnaire, consisting of both closed and open-ended questions. Of 15 questions, 5 related to the respondent’s socio-demographic data and 10 to the knowledge and opinions regarding preventive vaccinations with a focus on knowledge of the reasons for the introduction of a compulsory vaccination schedule, adverse events following immunization, and anti-vaccine movements. All questions were presented in Supplementary material 1. Completion of the questionnaire was anonymous and voluntary. The inclusion criteria for the study were age ≥ 18 years and informed consent to participate in the study given by a respondent. The questionnaires were collected using the snowball-sampling method, with the use of procedures that prevented the identification of the respondents. Questionaires were collected among the patients of the Department of Cardiology at the Faculty of Health Sciences in Katowice, their visitors, doctors, nurses, and paramedics working in Cardiology Department, and medical students who participated in the classes organized in the aforementioned Department. Questionnaires were also collected from the authors’ acquaintances. In the analysis of the results, all assessed parameters were presented, both in numerical and percentage values. Using the Chi^2^ test, the relationship between additional vaccinations intake and age, gender, education level, and the relationship with the medical professions were examined. For the purpose of mentioned analysis all participants were divided into 5 age groups (18–30, 31–40, 41–50, 50–60, >60 years). The members of the “medical professions” group were doctors, nurses, paramedics, and medical students. In all cases, the null hypothesis negated the existence of the relationship between the above-mentioned qualitative variables. For statistically significant values, the Cramer's V coefficient was calculated using the *Statistica 13.1* software, also used to calculate the Chi^2^ statistic value. The critical level of significance in all analyses was set at *p* < 0.05.

## Results

### General characteristics of the participants

The general characteristics of the participants are presented in Table 1.

**Table 1:** General characteristics of the participants

Participants (*n* = 700; 100%)				
**Variable**			**n**	**%**
**Sex**	Female		500	71.43
	Male		200	28.57
**Place of residence**	City		612	87.43
	Village		88	12.57
**Level of education taking into account participants’ sex**	Primary	F	16	2.29
		M	18	2.57
	Secondary	F	260	37.15
		M	102	14.57
	Higher	F	224	32.00
		M	80	11.43
**The existence of a relationship with the medical professions**	Yes		252	36.00
	No		448	64.00

Explanation of abbreviations: *n* number of participants, *F* female, *M* male

Almost 90% of the respondents lived in the cities, and over 70% were women, the majority of whom had secondary education. The smallest group, both among women and men, were individuals with primary education.

### Compulsory vaccinations

The characteristics of the participants, including the implementation of the vaccination schedule, are presented in section A of Table 2.

**Table 2:** Statements regarding compulsory vaccinations, the occurrence of adverse events after immunization among respondents, and anti-vaccine movements taking into account the number of received responses

Statements regarding:	n	%
**A.** **Characteristics of the participants taking into account the implementation of the vaccination schedule**		
I completed all compulsory vaccinations described in the vaccination schedule in force during my childhood	606	87.00
I did not complete all compulsory vaccinations described in the vaccination schedule in force during my childhood	26	4.00
I do not know if I have completed all compulsory vaccinations from the vaccination schedule in force during my childhood	66	9.00
**B.** **Characteristics of the participants including the occurrence of adverse events following immunization confirmed by a doctor**		
Yes, I had adverse events following immunization confirmed by the doctor	30	4.29
I have never had adverse events following immunization confirmed by a doctor	586	83.71
I do not know, if I ever had adverse events following immunization confirmed by the doctor	84	12.00
**C.** **Characteristics of the participants taking into account their knowledge about the activity of anti-vaccination movements in the public space**		
I know that there are anti-vaccination movements in the public space	676	96.57
I do not know that there are anti-vaccination movements in the public space	24	3.43
**D.** **Characteristics of the participants including support for anti-vaccination movements**		
I support anti-vaccination movements	30	4.29
I do not support anti-vaccination movements	670	95,71

Explanation of abbreviations: *n* number of participants

Almost 90% of respondents declared that they had complied with the complete compulsory vaccination schedule in force during their childhood.

**Fig. 1:**
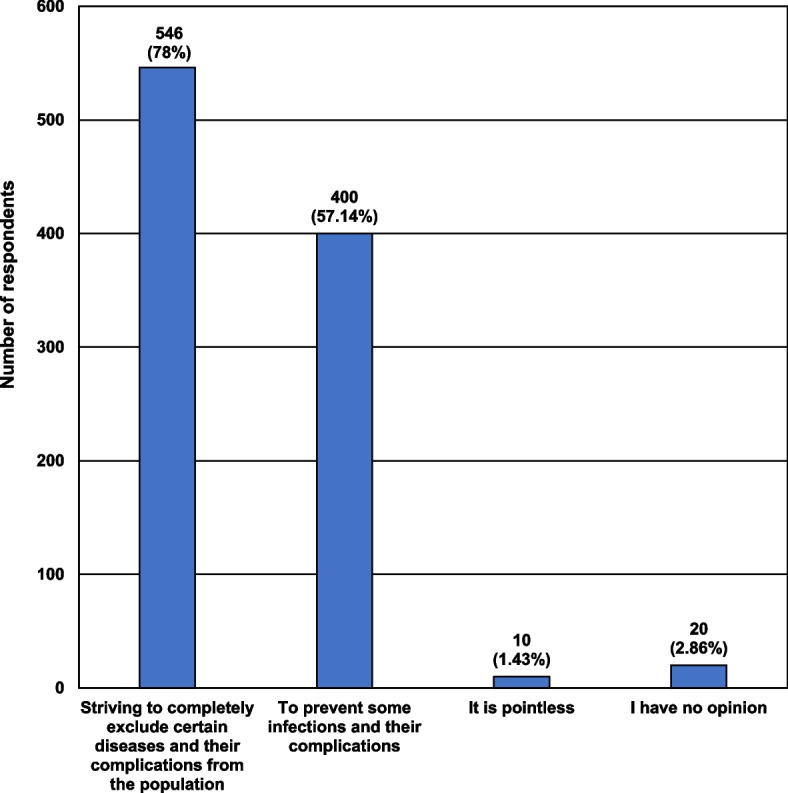
Characteristics of the participants taking into account their opinions about the purpose of introduction of a compulsory schedule of preventive vaccinations

The characteristics of the respondents, including their knowledge of the purpose of introducing a compulsory vaccination schedule, are presented in Fig. 1.

Almost 80% of participants believed that the main reason for the development of the vaccination schedule was the desire to completely exclude certain diseases and their complications from the population. Unfortunately, several respondents argued that its creation was pointless.

### Additional vaccinations

Characteristics of the participants, including an attempt to link the implementation of at least one additional vaccination with age, sex, education level, and the relationship with medical professions are presented in Table 3. Table 4 presents the most frequently performed additional vaccinations by the participants from the study.

**Table 3:** Characteristics of the participants including an attempt to link the implementation of at least one additional vaccination with age, gender, education level, and relationship with medical professions

Participants (*n* = 700; 100%)								
Performing at least one additional vaccination	**Yes (*n* = 324; 46.29%)**		**No (*n* = 376; 53.71%)**		**Σ** **n (%)**	***χ***^**2**^	***p***	**φ**_***c***_
Number of participants (n; %)	***n* = 324 (100%)**		***n* = 376 (100%)**					
Data/Variable	**n**	**%**	**n**	**%**				
Age (years)								
18–30	146	45.06 (40.78)	212	56.38 (59.22)	358 (100)	22.262	0.002^*^	0.18
31–40	20	6.17 (32.26)	42	11.17 (67.74)	62 (100)			
41–50	114	35.19 (58.76)	80	21.28 (41.24)	194 (100)			
51–60	14	4.32 (50.00)	14	3.72 (50.00)	28 (100)			
>60	30	9.26 (51.72)	28	7.45 (48.28)	58 (100)			
Sex								
Females	238	73.46 (47.60)	262	69.68 (52.40)	500 (100)	1.216	0.270	-
Males	86	26.54 (43.00)	114	30.32 (57.00)	200 (100)			
Level of education								
Primary	12	3.70 (35.29)	22	5.85 (64.71)	34 (100)	11.074	0.004^*^	0.13
Secondary	150	46.30 (41.44)	212	56.38 (58.56)	362 (100)			
Higher	162	50.00 (53.29)	142	37.77 (46.71)	304 (100)			
Relationship with medical professions								
Yes	126	38.88 (50.00)	126	33.51 (50.00)	252 (100)	2.185	0.139	-
No	198	61.11 (44.20)	250	66,49 (55.80)	448 (100)			

Explanation of abbreviations: *n* number of participants, *χ*^2^ Chi2 value, φ*c* Cramer's V coefficient value

^*^statistically significant at *p* < 0.05

**Table 4:** Characteristics of the participants taking into account the most frequently performed additional vaccinations by the respondents

Participants (*n* = 700; 100%)		
**Vaccination against:**	**n**	**%**
Influenza	200	28.57
Hepatitis type A	114	16.29
Smallpox	60	8.57
*Human Papillomavirus (HPV)*	34	4.86
Tick-borne encephalitis	16	2.29
Rotaviruses	14	2.00
*Neisseria meningitidis*	12	1.71

Explanation of abbreviations: *n *number of participants

More than half of the participants have never had any additional vaccination. Among the participants performing additional vaccinations, the largest group was between 18 and 30 years of age. A statistically significant correlation was found between performance of additional vaccinations and age (*p* = 0.002) and education level (*p* = 0.004). About 1/3 of participants were vaccinated against influenza. The least number of respondents were vaccinated against rotaviruses and *Neisseria meningitidis*.

### Adverse events following immunization

The characteristics of the participants, including their knowledge about adverse events following immunization, are presented in Table 5.

**Table 5:** Characteristics of the participants including their knowledge about possible adverse events following immunization

Participants (*n* = 700; 100%)		
**Symptoms/ events:**	**n**	**%**
Increase in body temperature (>40 °C)	658	94.00
Difficulty breathing and/or shortness of breath	396	56.57
Enlarged lymph nodes	268	38.29
Fainting and/or disturbed consciousness	236	33.71
A child's cry lasting minimally 3 h occurring within 2 days after vaccination	216	30.86
Seizures	174	24.86
Heart arrhythmia	142	20.29
Motor paresis	98	14.00
Autism	54	7.71
Loss of sight	52	7.43
Speech disorders	44	6.29
Vomiting with bile	40	5.71
Presence of blood in the stools	34	4.86

Explanation of abbreviations: *n* number of participants

Almost 95% of the participants correctly classified the increase in body temperature as a adverse event following immunization, while it was shocking that there was a group including autism in them.

Characteristics of the participants, including the presence of physician-confirmed adverse events following immunization, are presented in section B of Table 2.

Only about 5% of respondents experienced adverse events to the vaccine, which were confirmed by a doctor.

### Anti-vaccination movements

The characteristics of the participants, taking into account their knowledge about the activity of anti-vaccination movements, are presented in section C of Table 2. Section D of Table 2 presents the characteristics of the participants taking into account their support for abovementioned movements.

Almost the entire surveyed group knew about the activities of anti-vaccination movements in public space, and as many as 30 participants declared their support for them.

**Fig. 2:**
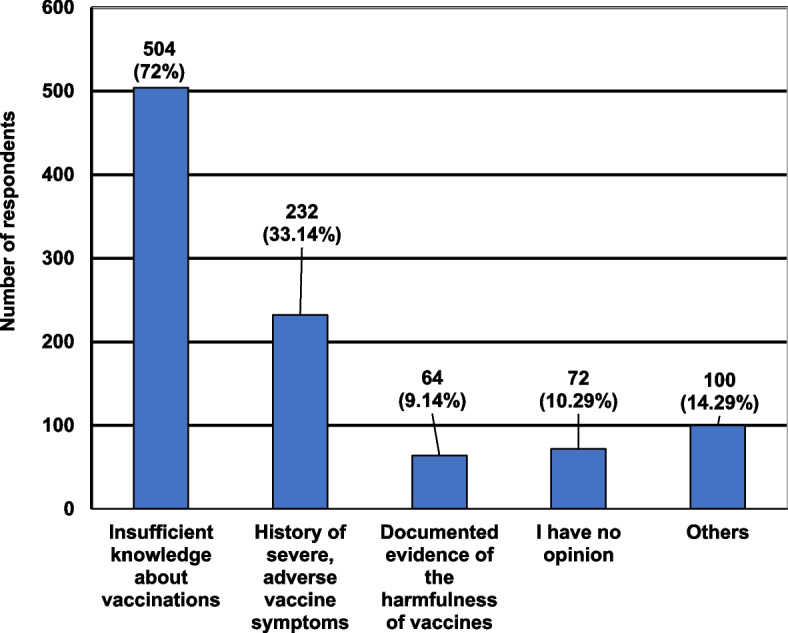
Characteristics of the participants taking into account their opinions about the reasons for the emergence of the anti-vaccination movements

Figure 2 presents the characteristics of the participants, taking into account their responses about the possible reasons for the emergence of anti-vaccination movements.

Over 70% of participants indicated that the main reason for the emergence of anti-vaccination movements was lack of sufficient knowledge about vaccinations. Less than 10% chose the presence of "Documented evidence of the harmfulness of vaccines". In the “Others” category respondents added: access to untested knowledge, fashion, ignorance on the part of the medical community, religious fundamentalism and sectarianism, an attempt to explain one’s child’s illness, and the stupidity of a society that believes in pseudoscientists theories.

**Fig. 3:**
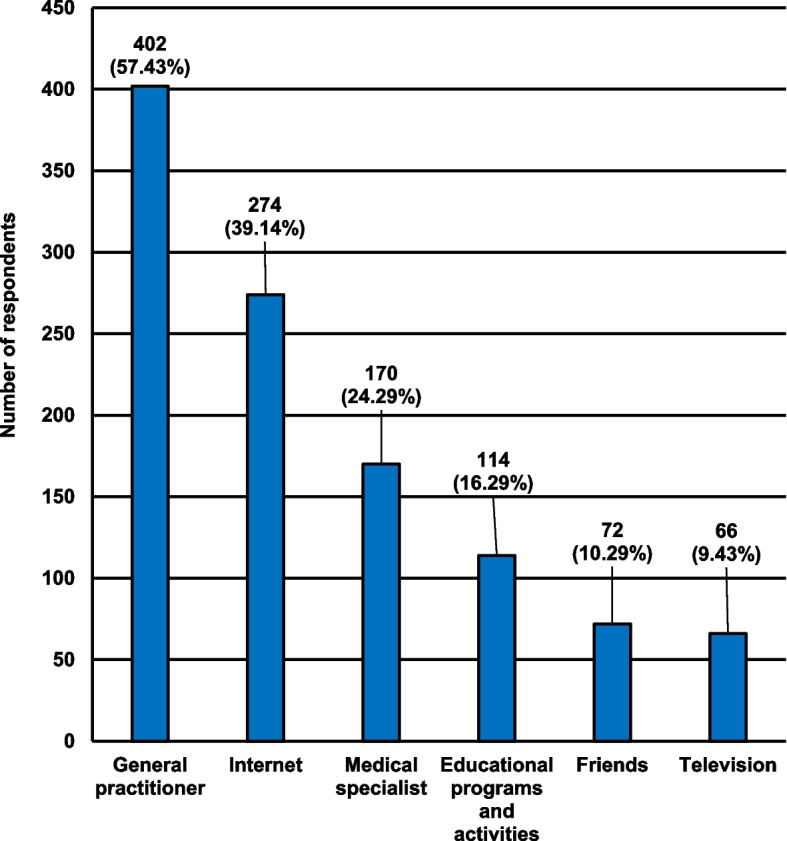
Characteristics of the participants including the preferred sources of obtaining information regarding vaccinations

The characteristics of the participants, taking into account the preferred sources of knowledge regarding vaccinations, are presented in Fig. 3.

The participants most willingly obtained information about preventive vaccinations from the general practitioner.

## Discussion

The presented results demonstrate what a challenge the topic of immunization is for adults and how much needs to be done in terms of public knowledge on the discussed subject. Almost 90% of the respondents had completed all compulsory vaccinations. This is extremely positive information because, as the data of the Polish National Institute of Public Health has revealed, the number of people who avoid compulsory immunization is increasing every year. In the period from January 1 to October 31, 2019, 44 475 waivers from the above-mentioned vaccinations were recorded in Poland. In the same period, in 2020, 50 575 cases were already registered [7].

The respondents stated that the main reason for the development of the vaccination schedule was the desire to completely exclude certain diseases and their complications from the population. Vaccination will prevent the spread of disease in the form of a pandemic that affects a large part of the population and contribute to the development of immunity that protects against the disease. Individual and group immunity can be distinguished [8, 9]. Herd immunity is achieved due to the high percentage of people vaccinated. It reduces the risk of unvaccinated people, which includes people who are not vaccinated due to age or medical indications, e. g. congenital immunity disorders or certain neoplastic diseases, but also people who deny the effectiveness of the procedure in question.

Only 46% of the respondents declared that they had performed at least one additional vaccination. A statistically significant correlation was found between the implementation of at least one additional vaccination and participants age (*p* = 0.002) and education level (*p* = 0.004). It is also disturbing that among people declaring the existence of a relationship with medical professions, the same number of respondents did and did not perform at least one additional preventive vaccination. The most frequently performed additional vaccinations included those against influenza (28.57%) and hepatitis type A (16.29%). In the studied group, the percentage of additional vaccination against influenza was much higher than in the general population of Poland. According to the data of the National Institute of Public Health, only 4.12% of Poles were vaccinated against influenza in the 2019/2020 season, and 3.9% in the previous season. Statistical data showed that in the past in Poland the highest level of vaccination was recorded in the 2001/2002 season—10.57%. Since then, the percentage of people vaccinated has continued to decline [10, 11]. It is certain that the number of people vaccinated against influenza will decrease more because of the emerging fake news about the harmfulness of vaccination, and also because of the current SARS-CoV-2 virus pandemic reducing the availability of the influenza vaccine.

The most frequently reported adverse events after vaccination were: an increase in body temperature (> 40 °C)—94%, breathing difficulties and dyspnoea—56.57%, and enlargement of lymph nodes—38.29%. Almost 8% of respondents classified autism as an adverse vaccine reaction. In the studies carried out by Zarobkiewicz M. et al., the most common adverse events after vaccination given by their respondents were: fever—36.29%, pain, swelling, and redness of the injection site—27.42%, allergic reaction—18% and rash—13.2%. The less frequently chosen responses were: weakness (10.32%) and anaphylactic shock (9.74%) [12]. In the group studied by Jurgiel J. et al., people indicated autism as an undesirable vaccine reaction over 10 times more often than the results from own research. As many as 81% of respondents in the above-mentioned study classified autism as an adverse vaccine reaction. The results of the studies by the authors cited above have also shown that adverse vaccination events are among the main reasons for vaccination concerns [13].

In the study group, the doctor found the occurrence of an undesirable vaccine reaction in only 30 respondents. According to the data of the National Institute of Public Health in Poland, in 2018 only 3,809 cases of adverse vaccine events were reported, of which 3,639 were classified as adverse events following immunization. Of the 3,639 cases mentioned, only 11 were classified as severe adverse events following immunization. The calculated percentage of severe adverse events following immunization based on the cited data of the National Institute of Public Health was much lower (approximately 0.3%) than that reported among the members of the study group [14, 15].

Almost 97% of the respondents knew about the activities of anti-vaccination movements. This is a much higher result than that obtained by Zarobkiewicz M. et al., who showed in their study conducted in a group of 1,386 students of medical and non-medical faculties that only 42.64% of respondents were aware of the presence of these movements [12].

Of the 700 surveyed participants, 30 were in favour of the anti-vaccination movements. There are no exact statistics on the number of individuals denying the effectiveness of vaccination in Poland and around the world. Research conducted in 2020 by the Center for Countering Digital Hate showed that since 2019 as many as 31 million people have liked Facebook profiles related to anti-vaccine movements, and over 17 million people subscribe to YouTube channels on this topic. Since 2019, the number of people with an interest in this problem has increased by at least 8 million [16]. The presented situation is extremely worrying, especially since the world is still struggling with the problems and consequences of the prevailing SARS-CoV-2 pandemic. To prevent the further development of anti-vaccination movements, the Strategic Advisory Group of Experts on Immunization Working Group on Vaccine Hesitancy at World Health Organization conducted a literature review and proposed a set of recommendations addressed to health professionals from all member states [17]. Recommendations are divided into three categories: the need to better understand the reasons for hesitating to take vaccines (1), deal with the structures of anti-vaccine organizations, and develop organizational capacity for health care to increase vaccine acceptance at the global, national, and local levels (2), focus exchange of lessons learned, effective practices from different countries and backgrounds, and the development, validation, and implementation of new tools to tackle vaccine abandonment (3) [18].

It may also be helpful to understand the reasons behind the development of these movements in combating anti-vaccination beliefs. The respondents mentioned the following as the main reasons for their creation: lack of sufficient knowledge about vaccinations, access to unproven knowledge, religious fundamentalism, as well as sectarianism, and trust in content promoted by pseudoscientists theories. Among the reasons for withholding vaccinations in children by 120 parents, the study by Jaroszewska K. et al. mentioned: concerns about the safety of preventive vaccinations (55.8%) and their effectiveness (49%) [19]. Studies conducted on the Italian population by Giambi C. et al. showed that the reasons for abandoning these vaccinations were: no recommendation from the paediatrician to fully vaccinate the child, receiving conflicting opinions about vaccinations, and meetings with the parents of children, who experienced severe side effects and treating children with traditional methods of treatment [20].

Both the results of own research and those cited in the discussion clearly show that the respondents lack knowledge in the field of vaccination, which should be improved through the implementation of educational programs and social and promotional campaigns. In the process of shaping the opinion and social knowledge about vaccination the role of members of the health care system, especially family doctors who should using appropriate arguments encourage patients to perform preventive vaccinations, is extremely important. The results of our research have shown that the doctors mentioned above are the most frequently chosen source of knowledge about vaccinations. The second most frequently chosen by the respondents’ source of knowledge about vaccines was the Internet. The available studies also confirm a similar trend not only in the Polish population but also in the world. The research by Yui Kwan Chow M. et al. showed that one of the most important sources of information on vaccinations for the inhabitants of Australia were family doctors [21]. Also, a study by Jaroszewska K. et al., conducted on a group of 130 people, showed that the main source of information about the benefits of immunization was the family doctor, and the second most frequently chosen source of knowledge by the respondents was the Internet [22]. Similar results were obtained in research by Rogalska J. et al., which included 1045 respondents. The aforementioned scientists showed that the family doctor was the main source of knowledge about vaccinations for both urban and rural residents. In the cited study, the Internet was the third most frequently chosen source of knowledge among city inhabitants, and the fifth most popular among rural area inhabitants [23].

To spread their postulates, anti-vaccine movements members use the Internet as a fast and widely available tool enabling easy access to information, but not always knowledge, which poses a real threat to public health. Despite the enormous amount of proven knowledge available on the Internet, much of the information on vaccination is not based on scientific evidence, but only on opinions, overheard comments, and out-of-context passages from members of the health care system and scientists. It is an ideal basis for representatives of the discussed movements to propagate false and harmful ideas. Worse still, untested, incorrect, and often untrue information negatively affects people who hesitate to get the vaccine or give it to their child. The research of the above-mentioned Jurgiel J. et al. showed that 33% of respondents gave up selected preventive vaccinations for their children after reading information about it on the Internet [13].

At this stage, the limitations of a study include a small group of adults, in which participants between 18 to 30 years predominated. Moreover, the majority of the sample were females which reduces the number of the opposite sex representatives and can influence obtained results. Finally, the majority of the population in study was enrolled in a hospital setting, thus the responses to the question concerning the preferred sources of knowledge and also the vaccination rates may represent a selection bias. Nonetheless, the study gives an inside into the general knowledge of Polish adults in the area of vaccinations. It has to be stated that vaccinations and the controversies they cause will not be resolved for many years. The results revealed that a problem for society is the knowledge of vaccinations, so further research should be conducted to examine the scope of vaccination omission, its causes and to determine which activities promoting the ideas behind Edward Jenner’s discovery over 200 years ago are best for current times. It is also important that the results presented have an impact on the increase of medical professionals’ interest in providing sufficient information about the need for vaccines administration to their patients.

## Conclusion

The degree of implementation of compulsory preventive vaccinations among participants was high, while additional vaccinations insufficient. The purposefulness of introducing a vaccination schedule was correctly determined by the majority of respondents, while their knowledge of adverse vaccination events and their types was incomplete. It is necessary to educate society on the benefits of preventive vaccinations and the damages caused by their avoidance, as well as the preparation of publicly available and controlled materials regarding discussed subject developed on Evidence-Based Medicine by specialists.

## Supplementary Information

**Additional file 1:**

## Data Availability

The datasets used and analysed during the current study are available from the corresponding author upon reasonable request.
