# Supplementary material for: Protective vaccinations in the control and prevention of infectious diseases—knowledge of adult Poles in this field. Preliminary results
Source: BMC Public Health. 2022 Dec 14;22:2342. doi: 10.1186/s12889-022-14821-2 (PMC9748885; doi:10.1186/s12889-022-14821-2)
Supplement: Supplementary file 1 — Additional file 1: [file 12889_2022_14821_MOESM1_ESM.docx]

**Supplementary material 1**

**Questions asked to the study participants**

**Suppl.1a.** Questions addressed to adults regarding described in the article topic.

| Asked questions: |
| --- |
| 1. Please, enter your age: ……………………………………. |
| 1. Choose your sex: 2. Female 3. Male |
| 1. Chose your place of residence:   a) City  b) Village |
| 1. Please, mark the level of your education: 2. Primary 3. Secondary 4. Higher |
| 1. Are you a doctor, nurse, paramedic or medical student? 2. Yes 3. No |
| 1. Please, choose the sentence which describes your vaccination status the best: 2. I have completed all compulsory vaccinations 3. I have not completed all compulsory vaccinations 4. I do not know if I have completed all compulsory vaccinations |
| 1. In your opinion, a compulsory vaccination schedule was introduced because:   a) Striving to completely exclude certain diseases and their complications from the population.  b) To prevent some infections and their complications  c) It was pointless  d) I have no opinion |
| 1. Have you ever received additional vaccinations? 2. Yes 3. No |
| 1. If you choose „*Yes*”, write down which one:   ……………………………………………… |
| 1. Which of the symptoms/reactions mentioned below may be classified as an unwanted post-vaccination reaction? 2. Autism 3. Loss of sight 4. Fainting and/or disturbed consciousness 5. Lymph nodes enlargement 6. Increase in body temperature (>40 °C) 7. Vomiting with bile 8. A child's cry lasting min. 3h occurring within 2 days of vaccination 9. Heart arrhythmia 10. Difficulty breathing and/or shortness of breath 11. Presence of blood in the stools 12. Motor paresis 13. Seizures 14. Speech disorders |
| 1. Have you ever had unwanted post-vaccination reactions confirmed by a doctor. a) Yes   b) No  c) I do not know |
| 1. Do you know that there are active anti-vaccine movements working in the society? 2. I know 3. I do not know |
| 1. Are you a supporter of anti-vaccine movements? 2. I support anti-vaccine movements 3. I do not support anti-vaccine movements |
| 1. Which of mentioned below, might be cause of the emergence of the anti-vaccine movement? 2. One insufficient knowledge about vaccinations 3. History of severe, adverse vaccine symptoms 4. Documented evidence of the harmfullness of vaccines 5. I have no opinion 6. Others (What kind?..........................). |
| 1. Which sources of informations about vaccinations do you prefere? 2. Internet 3. General practitioner 4. Other medical specialis 5. Educational programs and activities 6. Friends 7. Television |
